# Necrology

**Published:** 1898-09

**Authors:** 


### NECROLOGY.

A. L. HUNTER, V. S., Watkins, N. Y., died August 15, 1898, of pyæmia. The deceased was thirty-seven years old ; a graduate of the Ontario Veterinary College, class of 1885, and a member of the New York State Veterinary Medical Society. He enjoyed a good practice ; was active in political and agricultural interests of his district. He leaves a widow, but no children.

DR. L. M. BIGNELL, formerly of Philadelphia, Pa, but recently of Woodstown, N. J., where he was connected with one of the large breeding farms, died some weeks ago. Dr. Bignell was a graduate of the American Veterinary College, class of 1886, and while little known among association circles, he had gained for himself an enviable reputation as a successful practitioner.

